# Digital Surveillance of Mental Health Care Services in Saudi Arabia: Cross-Sectional Study of National e-Referral System Data

**DOI:** 10.2196/64257

**Published:** 2025-01-24

**Authors:** Abdullah A Alharbi, Nawfal A Aljerian, Meshary S Binhotan, Hani A Alghamdi, Ali K Alsultan, Mohammed S Arafat, Abdulrahman Aldhabib, Yasser A Alaska, Eid B Alwahbi, Mohammed A Muaddi, Ahmad Y Alqassim, Ronnie D Horner

**Affiliations:** 1 Department of Family and Community Medicine, Faculty of Medicine, Jazan University, Jazan, Saudi Arabia; 2 Medical Referrals Centre, Ministry of Health, Riyadh, Saudi Arabia; 3 Department of Emergency Medicine, King Saud bin Abdulaziz University for Health Sciences, Riyadh, Saudi Arabia; 4 Department of Emergency Medical Services, College of Applied Medical Sciences, King Saud bin Abdulaziz University for Health Sciences, Riyadh, Saudi Arabia; 5 King Abdullah International Medical Research Center, Riyadh, Saudi Arabia; 6 Department of Family and Community Medicine, College of Medicine, King Saud University, Riyadh, Saudi Arabia; 7 Department of Emergency Medicine, King Saud University, Riyadh, Saudi Arabia; 8 Department of Health Services Research and Administration, College of Public Health, University of Nebraska Medical Center, Omaha, NE, United States

**Keywords:** digital health, mental health, health policy, epidemiology, Saudi Arabia, SMARC, health care transformation, e-referral, Saudi Medical Appointments and Referrals Centre

## Abstract

**Background:**

Mental illness affects an estimated 25% of the global population, with treatment gaps persisting worldwide. The COVID-19 pandemic has exacerbated these challenges, leading to a significant increase in mental health issues globally. In Saudi Arabia, the lifetime prevalence of mental disorders is estimated at 34.2%, yet 86.1% of those with a 12-month mental disorder report no service use. To address these challenges, digital health solutions, particularly electronic referral (e-referral) systems, have emerged as powerful tools to improve care coordination and access. Saudi Arabia has pioneered the nationwide Saudi Medical Appointments and Referrals Centre (SMARC), a centralized e-referral system using artificial intelligence and predictive analytics.

**Objectives:**

This study aims to analyze Saudi Arabia’s novel nationwide e-referral system for mental health services, using SMARC platform data to examine referral patterns, and service accessibility. This study also aims to demonstrate how digital health technology can inform and improve mental health care delivery and policy making.

**Methods:**

This retrospective, cross-sectional study used secondary data from SMARC on 10,033 psychiatric e-referrals in Saudi Arabia during 2020‐2021. Referrals were assessed by patient sociodemographic variables, geographic data, and e-referral characteristics including date, type, bed type, and reason for e-referral. Descriptive statistical analyses identified referral patterns, while regression modeling determined predictors of external referrals to other regions.

**Results:**

Analysis of 10,033 psychiatric e-referrals revealed that 58.99% (n=5918) were for patients aged 18‐44 years, 63.93% (n=6414) were for men, and 87.10% (n=8739) were for Saudi nationals. The Western Business Unit generated 45.17% (n=4532) of all e-referral requests. Emergency cases were the most common type of referral overall, followed by routine inpatient and routine outpatient department referrals. However, in the Northern Business Unit, routine inpatient referrals were most frequent. Two-thirds of requests were for ward beds, while critical beds were rarely requested. “Unavailable subspecialty” was the primary reason for referrals across all regions. The utilization of the mental health e-referral system varied across regions, with the Northern Border and Albaha regions showing the highest rates, while Madinah, Eastern, and Riyadh regions demonstrated lower use. Temporal analysis showed almost similar monthly patterns in 2020 and 2021. There was an overall increase in referrals in 2021 compared with 2020.

**Conclusions:**

This pioneering study of mental health e-referrals in Saudi Arabia demonstrates how digital health transformation, particularly through an e-referral system, has significantly enhanced access to mental health services nationwide in Saudi Arabia. The success of this digital initiative demonstrates how digital health solutions can transform health care access, particularly in mental health services, offering a valuable model for other health care systems.

## Introduction

Mental illness remains a serious global health concern, with the World Health Organization estimating that it affects 25% of the population worldwide [1-3]. Significant treatment gaps persist globally, as around 70% of those needing mental health management lack access to care [4-8]. This high rate of untreated patients partly stems from specialist shortages and geographic barriers distancing patients from care [9,10], contributing to a major treatment gap. Furthermore, stigma and lack of mental health literacy continue to be significant barriers to seeking care, particularly in Middle Eastern countries [11].

The global landscape of mental health has evolved significantly in recent years, with the COVID-19 pandemic exacerbating existing challenges and creating new ones. Recent studies have shown that these gaps have widened during the COVID-19 pandemic, with a significant increase in mental health issues reported globally [12,13]. A meta-analysis estimated that the pandemic led to an additional 53.2 million cases of major depressive disorder and 76.2 million cases of anxiety disorders globally in 2020 alone [14]. These findings underscore the urgent need for scalable and accessible mental health interventions worldwide.

In response to these challenges, digital health solutions have emerged as powerful tools to improve coordination and access to mental health care. Electronic referral (e-referral) systems, in particular, have shown promise in reducing wait times, improving care coordination, and increasing access to specialist care [15,16]. The Kingdom of Saudi Arabia (KSA) has been an early pioneer in leveraging digital health through the nationwide Saudi Medical Appointments and Referrals Centre (SMARC). This centralized e-referral system was revamped and relaunched in 2019 to unify and streamline referrals across the country’s 13 administrative regions. The advanced SMARC platform uses artificial intelligence and predictive analytics to coordinate referrals between public and private hospitals, addressing previous fragmentation. As of 2019, the majority of governmental and private Saudi health care facilities are connected to SMARC, enabling efficient nationwide referral management—a major milestone in the KSA’s health sector digital transformation under Saudi vision 2030 [17,18].

Recent studies have highlighted significant challenges and opportunities to improve mental health care in Saudi Arabia. Estimates show that 34.2% of the general Saudi population experience a mental disorder during their lifetime [19]. Regarding depressive disorders, there was about a 60% increase in number of new cases from 1.42 million cases in 2011 to 2.28 million cases in 2021. The incidence rate also rose by around 23% during the same period, going from 4,913.71 to 6,039.77 per 100,000 people [20]. Despite this high prevalence, access to mental health care remains limited, with a national survey revealing that about 84% (597/711) of participants with a 12-month mental disorder reported no service use [21]. Barriers to care include low perceived need for help, cultural stigma, and attitudinal obstacles [22]. These findings emphasize the critical role that e-referral systems such as SMARC could play in addressing these challenges.

SMARC facilitates analysis of mental health patterns at a national scale, providing a unique opportunity for epidemiological and health services research on mental illnesses [1,2]. However, no large-scale studies have been conducted to date in the KSA, making such analysis a high priority [23]. This innovative study is the first to examine Saudi Arabia’s adoption of an e-referral system for mental health services, establishing digital monitoring of mental health care referrals. By analyzing national referral trends using SMARC platform data, it provides crucial insights into care patterns and resource distribution. The research showcases Saudi Arabia’s innovative use of technology to evaluate and enhance mental health service provision and accessibility. Our findings, derived from SMARC data, illuminate potential gaps in the mental health care system and could inform evidence-based policies and interventions. This aligns with the Saudi’s digital-driven health care transformation, offering valuable insights to optimize mental health care delivery throughout Saudi Arabia. Ultimately, this study contributes to the broader goal of improving mental health outcomes by leveraging technology to guide strategic health care decisions and resource allocation.

## Methods

### Study Design and Setting

This descriptive study of psychiatric health care delivery used data by the SMARC system between January 2020 and December 2021. The SMARC system is concerned with all e-referrals across the KSA. Unlike conventional referral systems, this system primarily serves e-referral between secondary, tertiary, and specialized health care providers. The SMARC system routinely collects an array of data concerning each e-referral request.

A descriptive approach was chosen for this study due to several key factors. First, this is the first comprehensive analysis of the Saudi national e-referral system for mental health services, necessitating an exploratory approach to establish baseline patterns and trends. Second, the descriptive nature of our study provides a crucial foundation for generating hypotheses that can guide future in-depth research. Third, given the novelty of the data source and the lack of prior comprehensive analyses in this context, a descriptive approach allows for a broad understanding of the current landscape of mental health referrals in Saudi Arabia. Finally, our descriptive findings offer immediate, actionable insights for policy makers and health care planners, which is particularly valuable given the urgency of addressing mental health needs in the region.

To initiate a referral request, a treating physician has to first identify the need and type of the referral, emergency or routine (both inpatient and outpatient). The Office of Coordination and Eligibility for Treatment, which is allocated in every hospital, digitally uploads the referral request at SMARC e-referral system. The Office of Coordination and Eligibility for Treatment can identify 3 potential receiving hospitals or can leave the task to the e-referral system.

**Figure 1.**
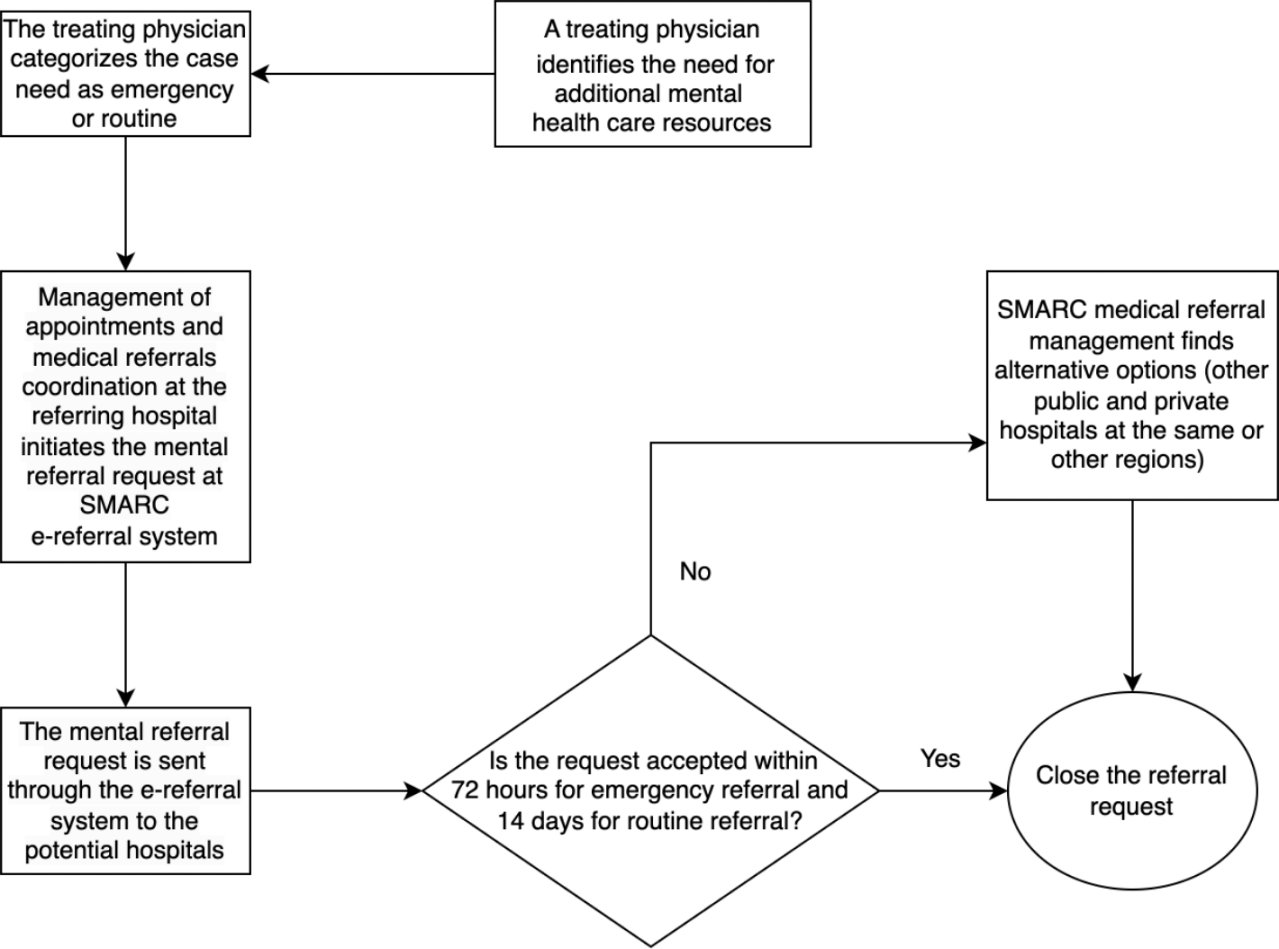
Flowchart depicting the process of initiating and accepting psychiatric e-referral requests through the SMARC in Saudi Arabia from January 2020 to December 2021. This cross-sectional study analyzed data on 10,033 psychiatric e-referrals across all regions of Saudi Arabia during this 2-year period. e-Referral: electronic referral; SMARC: Saudi Medical Appointments and Referrals Centre.

The referral request is sent, through the e-referral system, to the potential receiving hospitals for acceptance. The e-referral system has built-in time frames for requests to be accepted, depending on the referral type. For emergency requests, the system allows hospitals 72 hours to accept the request, while routine requests have 14 days. If the initially identified hospitals reject the request within the allotted time frame, further hospitals will receive the request for acceptance consideration. Once the allotted time frame elapses with no acceptance, the request will automatically be escalated to SMARC medical referral management team to secure an acceptance. The team will look at alternative options including other governmental and private hospitals, both at the same region and other regions if needed. During the process of referral initiation and acceptance, all patients continue receiving the necessary health care management at the referring hospital, to ensure stability, until being transferred to the receiving hospital. Figure 1 describes the process of mental referral request initiation and acceptance.

### Study Variables

All e-referral data provided by the SMARC system were used in this analysis, totaling 10,033 referrals. The variables included sociodemographic variables such as age, sex, and nationality of the patient with the e-referral request, as well as the patient’s administrative area of residence and business unit (BU) from which the request originated. BU refers to a geographical and administrative area designated for health care service delivery. The health care system comprises 5 BUs, each managed by a government-owned holding company. The units are distributed based on geographical coverage: Central BU includes Riyadh and AL Qassim; Western BU covers Makkah, Madinah, and Albaha; Eastern BU includes the Eastern region; Northern BU consists of Al-jouf, Northern Border, Tabuk, and Hail; and Southern BU includes Aseer, Jazan, and Najran. Each BU is tasked with managing and providing health care services within its respective regions.

Other variables pertain to the e-referral request itself, including the date, type, bed type, and reason. The type of e-referral includes routine outpatient department (OPD), routine inpatient, and emergency. Routine referrals (both OPD and inpatient) are those that do not require prompt intervention and are supposed to be accepted within 14 days. An emergency e-referral is a case requiring medical or surgical intervention within 72 hours to avoid mortality or morbidity. The bed type refers to OPD, ward bed, or critical bed. A critical bed indicates a case needing admission to the intensive care unit. Reasons for e-referral include unavailable specialty, unavailable physician, unavailable machine (meaning no tools), and unavailable bed (meaning no available bed at the hospital).

### Statistical Analysis

Descriptive statistics were calculated for all variables, including frequencies and percentages. To explore potential regional differences, we conducted cross-tabulations of each variable across the 5 BUs and performed *χ*² tests to assess associations. STATA (version 16; StataCorp LLC) was used for all analyses. Furthermore, we generated color-coded maps using ArcGIS software (ArcGIS 10.0; Environmental Systems Research Institute, Inc) to visually represent the distribution of e-referrals requests across different administrative areas.

### Ethical Considerations

This study was conducted in accordance with the Declaration of Helsinki and approved by the institutional review board of the Ministry of Health (MOH) (IRB log No. 23‐77-E; date: September 20, 2023). The requirement for individual informed consent was waived due to the retrospective nature of the study and the use of deidentified data. Patient privacy and confidentiality were strictly maintained throughout the research process. All data obtained from the SMARC system were anonymized prior to analysis. Access to the dataset was restricted to authorized research team members and stored on secure, password-protected servers. The study did not involve any direct contact with patients or any intervention that could affect their care. We confirm that no images or information that could lead to the identification of individual participants is included in this manuscript or any supplementary materials.

## Results

### Sociodemographic Characteristics of Psychiatry Patients With e-Referral Requests

Of the 10,033 patients with a psychiatry-related e-referrals during the study period, 58.99% (n=5918) were aged 18‐44 years, followed by those aged 45‐64 years (n=1869, 18.63%). Patients younger than 18 years or older than 65 years comprised relatively few requests. Nearly two-thirds of requests were for men (6414/10,033, 63.93%) compared with women (3619/10,033, 36.07%). The majority (8739/10,033, 87.1%) were Saudi nationals. Approximately 45% (n=4532) of all e-referral requests originated from the Western BU, while the other regions each generated around one-third or less than this proportion (Table 1).

**Table 1.** Sociodemographic characteristics of psychiatry patients with e-referral requests in Saudi Arabia from January 2020 to December 2021^a^.

Characteristics	Participants (N=10,033), n (%)
**Age (years)**	
<11	844 (8.41)
12‐17	617 (6.15)
18‐44	5918 (58.99)
45‐64	1869 (18.63)
>65	785 (7.82)
**Sex**	
Male	6414 (63.93)
Female	3619 (36.07)
**Nationality**	
Non-Saudi	1294 (12.90)
Saudi	8739 (87.10)
**Business unit**	
Central	1128 (11.24)
Eastern	1220 (12.16)
Western	4532 (45.17)
Northern	1603 (15.98)
Southern	1550 (15.45)
**Year**	
2020	4179 (41.65)
2021	5854 (58.35)

a This cross-sectional study analyzed data on 10,033 psychiatric e-referral requests across the 5 business units (Central, Eastern, Western, Northern, and Southern) in Saudi Arabia between January 2020 and December 2021. Data used were the data routinely collected by the Saudi Medical Appointments and Referrals Centre (SMARC) system. The SMARC system is concerned with all e-referral requests in Saudi Arabia.

### Sociodemographic Characteristics of Psychiatry Patients With e-Referral Requests According to BUs

Table 2 shows the sociodemographic characteristics of patients with a psychiatry-related e-referral request for each of the 5 BUs. In general, the patients being referred for psychiatric care in each BU were similar in characteristics to those identified for the population as a whole. These associations within regions were all significant at the *P*<.001 level.

**Table 2.** Sociodemographic profile of psychiatric e-referral patients across different business units in Saudi Arabia from January 2020 to December 2021^a^.

Characteristics	Central (1128/10,033, 11.24%), n (%)	Eastern (1220/10,033, 12.16%), n (%)	Western (4532/10,033, 45.17%), n (%)	Northern (1603/10,033, 15.98%), n (%)	Southern (1550/10,033, 15.45%), n (%)
**Age (years) (** ***P*** **<.001)**					
<11	72 (6.38)	130 (10.66)	341 (7.52)	121 (7.55)	180 (11.61)
12‐17	71 (6.29)	105 (8.61)	260 (5.74)	73 (4.55)	108 (6.97)
18‐44	704 (62.41)	643 (52.70)	2878 (63.50)	829 (51.72)	864 (55.74)
45‐64	230 (20.39)	253 (20.74)	799 (17.63)	334 (20.84)	253 (16.32)
>65	51 (4.25)	89 (7.30)	254 (5.60)	246 (15.35)	145 (9.35)
**Sex (*P*<.001)**					
Males	694 (61.52)	739 (60.57)	2998 (66.15)	998 (62.26)	988 (63.74)
Females	434 (38.48)	481 (39.43)	1534 (33.85)	605 (37.74)	562 (36.26)
**Nationality (*P*<.001)**					
Non-Saudi	107 (9.49)	106 (8.69)	658 (14.52)	189 (11.79)	234 (15.10)
Saudi	1021 (90.51)	1114 (91.31)	3874 (85.48)	1414 (88.21)	1316 (84.90)

a This cross-sectional study analyzed data on 10,033 psychiatric e-referral requests across the 5 business units (Central, Eastern, Western, Northern, and Southern) in Saudi Arabia between January 2020 and December 2021. Data used were the data routinely collected by the Saudi Medical Appointments and Referrals Centre (SMARC) system. The SMARC system is concerned with all e-referral requests in Saudi Arabia. Correlation between variables was examined through a *χ*² test, revealing a highly significant difference at a significance level of *P*≤.001.

### e-Referrals Characteristics of Psychiatry Patients by BU

Table 3 presents the results of psychiatry e-referral requests overall and for each of the 5 BUs. Out of the 3 types of psychiatry e-referrals , the most common type was for emergency cases and the least reported type was routine OPD e-referral. Emergency psychiatric e-referral was also the most common type for each BU with the exception of the Northern BUs, where routine inpatient e-referral was the highest. With regard to bed types, two-thirds of all requests were for ward beds; very few requests were for critical beds. Ward beds were also the most commonly requested type of bed across BUs. “Unavailable subspecialty” was the most commonly reported reason for the psychiatry-related e-referrals overall, whereas the “unavailability of a machine” was the least reported reason. Among each BUs, a similar pattern was observed for the reason for the e-referral.

**Table 3.** Characteristics of psychiatric e-referrals^a^ across different business units in Saudi Arabia from January 2020 to December 2021^b^.

Characteristic	Central (1128/10,033, 11.24%), n (%)	Eastern (1220/10,033, 12.16%), n (%)	Western (4532/10,033, 45.17%), n (%)	Northern (1603/10,033, 15.98%), n (%)	Southern (1550/10,033, 15.45%), n (%)	Total (N=10,033), n (%)
**e-Referral types (*P*<.001)**						
Routine OPD^c^	411 (36.44)	447 (36.64)	1297 (28.62)	326 (20.34)	437 (28.19)	2918 (29.08)
Routine inpatient	192 (17.02)	325 (26.64)	1473 (32.50)	675 (42.11)	410 (26.45)	3075 (30.65)
Emergency	525 (46.54)	448 (36.72)	1762 (38.88)	602 (37.55)	703 (45.35)	4040 (40.27)
**Bed type (*P*<.001)**						
OPD	409 (36.26)	447 (36.64)	1292 (28.51)	324 (20.21)	437 (28.19)	2918 (29.08)
Ward	709 (62.85)	750 (61.48)	3090 (68.18)	1224 (76.36)	1064 (68.65)	6828 (68.06)
Critical	10 (0.89)	23 (1.89)	150 (3.31)	55 (3.43)	49 (3.16)	287 (2.86)
**Reason of e-referral (*P*<.001)**						
Unavailable subspecialty	767 (68)	776 (63.61)	3197 (70.54)	1046 (65.25)	1107 (71.42)	6893 (68.70)
Unavailable physician	249 (22.07)	394 (32.30)	944 (20.83)	473 (29.51)	292 (18.84)	2352 (23.44)
Unavailable machine	26 (2.30)	34 (2.79)	97 (2.14)	61 (3.81)	96 (6.19)	314 (3.13)
Unavailable bed	86 (7.62)	16 (1.31)	294 (6.49)	23 (1.43)	55 (3.15)	474 (4.72)

a e-referral: electronic referral.

b This cross-sectional study analyzed data on 10,033 psychiatric e-referrals across the 5 business units (Central, Eastern, Western, Northern, and Southern) in Saudi Arabia between January 2020 and December 2021. Data used were the data routinely collected by the Saudi Medical Appointments and Referrals Centre (SMARC) system. The SMARC system is concerned with all e-referral in Saudi Arabia. . Correlation between variables was examined through a *χ*² test, revealing a highly significant difference at a significance level of *P*≤.001.

c OPD: outpatient department.

### Trends and Patterns of Psychiatry e-Referral Requests

**Figure 2.**
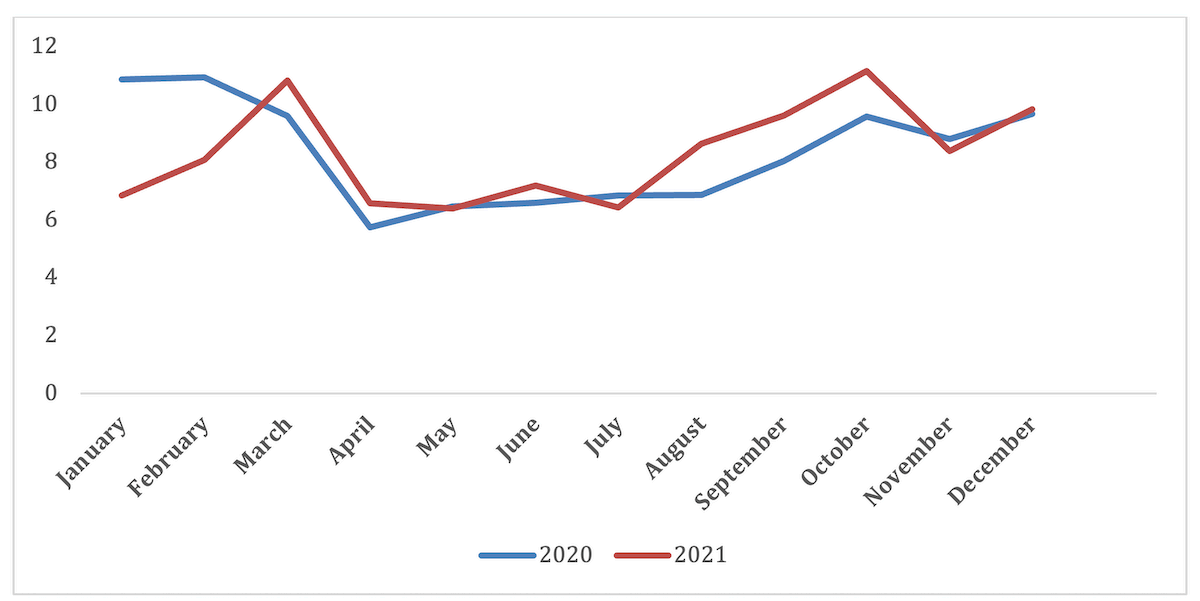
Monthly trend of psychiatric e-referrals in Saudi Arabia from January 2020 to December 2021, based on data routinely collected by the Saudi Medical Appointments and Referrals Centre (SMARC). This cross-sectional study analyzed data on 10,033 psychiatric e-referrals processed through the SMARC system across all regions of Saudi Arabia during this 2-year period. The line graph depicts the pattern of psychiatric e-referrals per month. e-Referral: electronic referral.

Figure 2 shows the trend of psychiatry-related e-referral requests across months separately for 2020 and 2021. For both years, the pattern was similar across the months between March and December, with slightly higher peaks for 2021. However, there is a clear discrepancy or divergence between years in the pattern of requests between January and March.

### Geographic Patterns of Psychiatric e-Referral Requests Across Different Administrative Areas in Saudi Arabia

**Figure 3.**
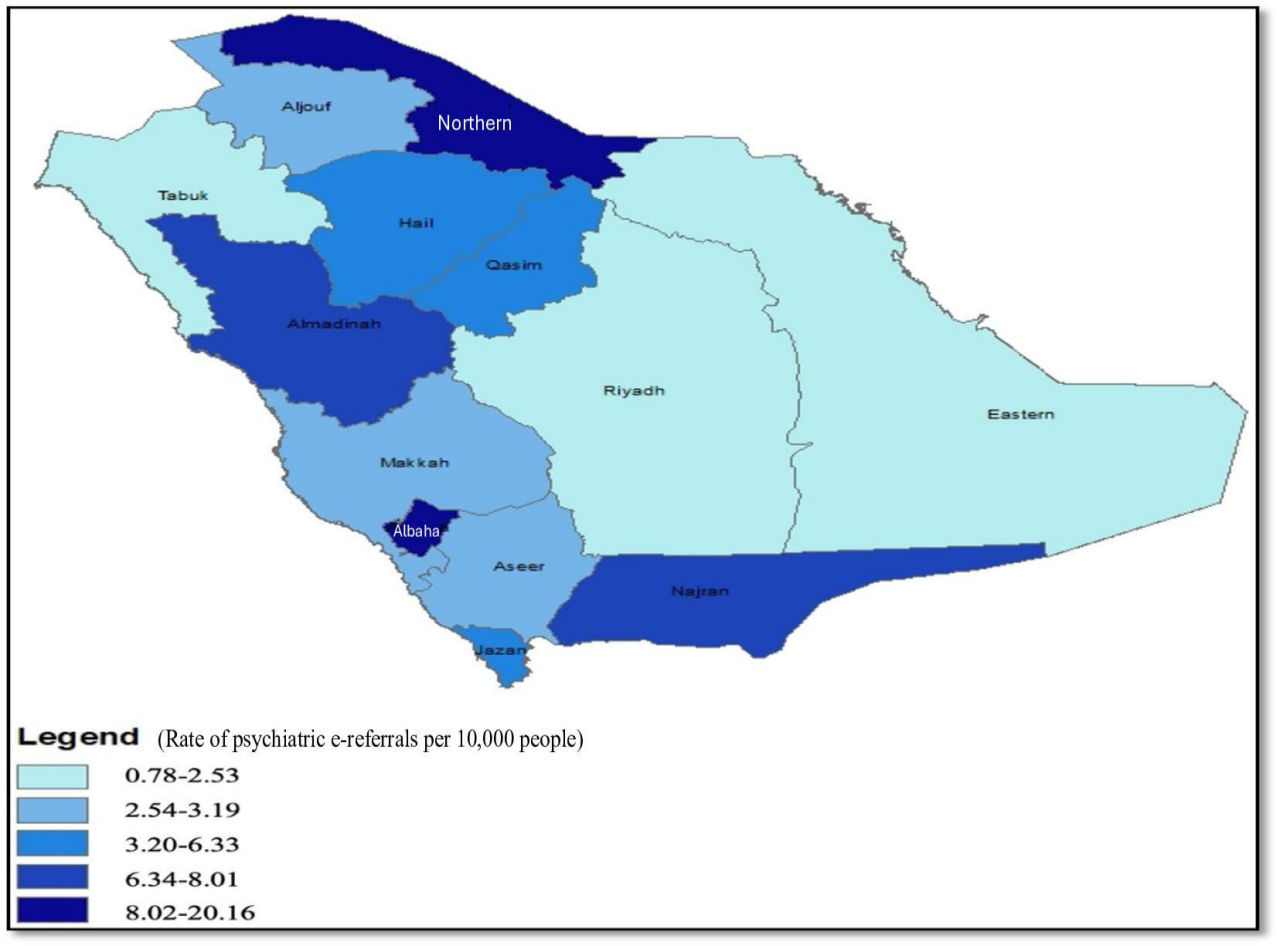
usGeographic distribution of psychiatric e-referral requests per 10,000 people across administrative regions of Saudi Arabia from January 2020 to December 2021, based on data from a cross-sectional study of 10,033 e-referrals. The map, developed using ArcGIS, depicts the rate of psychiatric e-referrals per 10,000 people in each region, with darker shades of blue indicating higher rates (upper quantiles) and lighter shades indicating lower rates (lower quantiles). The map highlights regional variations in the utilization of the Saudi Medical Appointments and Referrals Centre for psychiatric referrals during the study period. e-Referral: electronic referral.

Figure 3 shows the administrative regions’ rates of e-referral requests per 10,000 people. The Northern Border and Albaha regions had the highest rates and fell into the highest quantile. In contrast, the Madinah, Eastern, and Riyadh regions had the lowest rates of e-referral requests per 10,000 people. See Table S1 in Multimedia Appendix 1 for more details on the rates by region.

## Discussion

### Principal Findings

This national study primarily aims to analyze psychiatric e-referral patterns across Saudi Arabia, while also providing an overview of the sociodemographic and clinical characteristics of mental health referrals across different BUs under the new health care transformation, emphasizing regional needs. The analysis is enabled by Saudi Arabia’s investments in advanced digital health systems, the nationwide e-referral platform SMARC, allowing examination of mental health referral trends at an unprecedented scale. Our findings indicate that e-referrals predominantly involved Saudi nationals, males, and working-age adults (aged 18‐44 years), with most referrals from the Western BU and an increase in 2021 compared with 2020. Moreover, among these mental health referrals, emergency referrals were the most common e-referral type, ward beds being the most requested, and the essential cause was the lack of specialized providers.

Males were referred more frequently than females in the e-referral data. More than half of referrals were for males, highlighting a considerable gender imbalance that warrants further investigation into the underlying causes. The higher male referral rate may stem from men delaying or avoiding mental health treatment, leading to increased severity when finally referred, as untreated conditions may worsen over time [24,25]. This is supported by our findings where more emergency referrals were initiated for males compared with females, indicating the potential delay in seeking help (Table S2 in Multimedia Appendix 1). This delay in help-seeking among men has been linked with a higher risk of suicide [26], potentially explaining the higher referral rate. Broader literature supports this trend, reporting higher psychiatric emergency referrals and admission rates to psychiatric units for males compared with females [27,28].

The gender variation in referrals could also be attributed to differences in the prevalence of mental disorders. Males are more frequently associated with drug and substance use, disruptive disorders, schizophrenia, and a higher risk of suicide [29-33], often requiring more intensive resources. Conversely, females tend to experience higher rates of depression, anxiety, and somatic complaints [6,34], which can often be managed without referral. Studies in Saudi Arabia reflect similar patterns, with substance use and disruptive disorders more common in males, and anxiety and depressive disorders more prevalent among females [19,35].

These differences may have influenced the referral rates due to the availability of the required health care resources for each condition, since a referral is initiated when the needed resources such as psychiatry subspecialty are not available at the patient’s initial point of care. Nevertheless, these findings do not assess the general mental health care access in Saudi Arabia. Despite limited data of mental health care access, two-thirds of patients with depression who accessed primary health care were females [36], suggesting that both males and females can access mental health care services. However, our data lack detailed diagnostic information, necessitating further research to confirm these speculations and develop evidence-based recommendations.

The peak referral rate among 18‐ to 44-year-olds indicates a need for youth-focused mental health services and preventive strategies. Previous research found that this age group has a high mental illness burden [37,38]. However, responsibilities, such as work-life balance, finances, and caregiving [39,40], may contribute to increased mental health issues as reflected in higher referral requests among this age group.

Monthly referral patterns showed a bimodal distribution with peaks in March and October (Figure 1). This pattern suggests that there may be specific factors driving increased demand for mental health services during these months. These trends are likely due to the COVID-19 pandemic, , as pandemic waves escalated in Saudi Arabia during these months [41]. Isolation, anxiety, and stress likely increased emotional health needs during the pandemic, while lockdowns reduced access to in-person mental health services [42]. Social distancing, job losses, and fear of contracting the virus may have increased depression and anxiety [43]. During pandemic peak periods, mental health facilities may have had to limit in-person appointments or close, delaying referral processes [12].

Regional variation in referrals warrants more research. The Western region had the highest proportion of referrals, possibly due to its higher population density and mental health resources versus other regions. The Western region has more psychiatric manpower and hospital beds per capita compared with the Central region. For example, the Western region has a slightly higher proportion of psychiatrists (2.88 per 100,000 people vs 2.68 per 100,000 people) and psychiatric beds (1.32 per 10,000 people vs 1.03 per 10,000 people) than the Central region (Table S1 in Multimedia Appendix 1). This greater capacity may enable more internal referrals. Distinguishing internal versus external referrals by region could elucidate the observed patterns.

### Saudi Arabia National Health Care System

Saudi Arabia’s health care system serves 34 million people across 13 regions through a mix of public and private facilities. Aligning with Vision 2030 reforms, the system aims to improve quality, efficiency, and value by integrating the public and private sectors [44,45]. This involves establishing 5 new BUs to manage the regions alongside national insurance companies, overseen by the MOH and new insurance centers.

Fortunately, under Vision 2030, the MOH is pioneering a new health care paradigm focused on enhancing social, mental, and physical well-being through a patient-centered model of care (MOC) [46]. Structured around 6 systems, including mental health within chronic care [47], the MOC seeks to tailor health care delivery to individual needs. The MOH is developing a national mental health strategy following the MOC’s principles of patient-centered and integrated services starting at the community level [23]. This addresses care gaps and establishes accessible, quality services, supported by the new “National Committee for Mental Health Promotion” [48].

### Applying e-Referral Findings to Enhance Mental Health Systems

International and national studies have highlighted the difficulty in measuring quality via health care utilization rates and the significant variation in service quality among countries, cities, hospitals, and even among BUs [44,49,50]. In our study, mental health referrals varied significantly across BUs by demographic and clinical characteristics. Referral rates for those younger than 18 years peaked in the Eastern BU and then Southern BU, ages 18‐44 years in the Western BU, and older adults in the Northern BU. Increasing child psychiatrists, youth mental health programs, school initiatives, and young adult services are suggested for these BUs [51]. Female referrals were slightly higher in the Eastern BU, mirroring the overall elevated referral rate for females found in a prior study examining total referral patterns across all health conditions, not just mental health specifically [44]. It is recommended to tailor gender-specific services for women and develop outreach programs to encourage support-seeking, especially for mental health issues associated with women such as pre- and postpartum mental issues [52,53] across all BU regions, particularly those with high referrals. These findings provide actionable evidence for decentralizing and customizing mental health services for local needs.

The Southern BU had double or triple the referrals due to unavailable machines compared with other BUs, aligning with a previous study showing that the Southern BU had the highest referral rate for all health care scopes [44]. However, in this study, the Central BU had the highest referrals due to unavailable beds. Referrals from unavailable services or psychiatrists were similar across BUs. OPD referrals were highest in the Central and Eastern BUs, while routine inpatient referrals were highest in the Northern BU, and emergency conditions referrals were highest in the Central and Southern BUs. To enhance the distribution of mental health resources, a system could be implemented to standardize essential services and psychiatrists across BUs [54]. Infrastructure investments, increased psychiatric beds, and expanded outpatient and inpatient services may be especially beneficial for BUs with high e-referrals rates.

The World Health Organization advocates integrating mental health into primary care to enhance access, reduce stigma, promote social integration, and build health care capacity for mental disorders [55]. Aligning with this, Saudi Arabia has expanded mental health training for primary care physicians and general practitioners to provide community-based services [56,57]. General practitioners in primary care settings typically address minor mental health disorders through counseling, while more complex cases require specialist care [23]. Ongoing training is needed to enhance care quality by mental health professionals. Nonetheless, primary care plays a key role in early detection of mental health issues and appropriate e-referral to specialized care based on condition severity and complexity [56].

### Policy Implications

Our analysis revealed regional differences in Saudi Arabia’s mental health e-referral patterns. We propose several policies to improve e-referral rates based on our findings. First, invest in preventive measures, including reducing stigma through awareness campaigns, to decrease the prevalence and severity of mental health issues. This can be achieved through education, counseling or support access, physical activity promotion, stress management programs, community networks, early intervention for at-risk groups, and encouraging early help-seeking before severity worsens, potentially decreasing late-stage e-referral [58-61]. Second, strengthening primary care providers’ mental health training could enable earlier identification and management [62]. Integrated care models with mental health professionals also facilitate connections across levels, aligning with Saudi Arabia’s new health clusters unifying primary, hospital, and specialty care [63,64]. Third, expand telehealth and e-mental health tools to increase access to mental health services [65,66]. Fourth, grow the qualified mental health workforce through education and training investments to expand capacity [67]. Overall, multifaceted strategies focused on training, integration, technology, capacity building, and stigma reduction could enhance Saudi Arabia’s mental health e-referral system and outcomes.

It is important to note that this study was conducted during the peak of the COVID-19 pandemic, which may have influenced the observed referral patterns. While this timing introduces potential biases, it also provides a unique opportunity to understand mental health service utilization during a global crisis. Our findings serve as a crucial baseline for future comparisons with postpandemic periods. This research not only contributes to national policy making but also adds to the limited global literature on mental health service evaluations during extraordinary circumstances. Future studies should aim to compare these findings with postpandemic data to fully understand the long-term impacts of the crisis on mental health referral patterns and service delivery. Such comparisons will be invaluable in developing resilient mental health systems capable of adapting to both routine and crisis scenarios.

### Limitations

This study’s limitations stem from its reliance on secondary e-referral data, which lack detailed clinical information and comprehensive mental health infrastructure data across regions. This restricted our ability to conduct in-depth analyses of referral trend drivers and fully evaluate care access gaps. The study’s timing during the peak of the COVID-19 pandemic may have introduced biases in referral patterns, potentially limiting the generalizability of findings to noncrisis periods. However, this unique context also offers valuable insights into mental health service needs and utilization during global emergencies. We acknowledge that e-referral data alone cannot capture the full complexity of mental health issues in Saudi Arabia, and the scarcity of comparable research in Saudi Arabia limited contextualization of our findings. To address these limitations, we propose future research directions including longitudinal studies incorporating detailed diagnostic information, cultural factors, and resource availability data. Future qualitative research could explore whether regional differences exist in mental health attitudes and help-seeking behaviors. Furthermore, integrating socioeconomic data may provide further insights into factors influencing health care utilization. Collaborative research involving religious scholars would provide valuable insights. Postpandemic research is also crucial to determine how referral patterns have evolved. These comprehensive approaches would not only address current study limitations but also offer a more nuanced understanding of mental health in Saudi Arabia, informing targeted interventions and policy development to improve mental health care delivery and access nationwide.

### Conclusions

This nationwide study provides the first in-depth analysis specific to mental health e-referral patterns in Saudi Arabia, enabled by the large study sample of more than 10,033 referrals. Mental e-referrals predominantly involved Saudi nationals, males, and working-age adults (aged 18-44 years), with most referrals from the Western regions and a notable increase in 2021 compared with 2020. Emergency referrals were most common, with ward beds being the most requested due to a lack of mental health specialty services. Regional variations in e-referral patterns were observed. Given the widespread challenges in mental health care globally, these findings emphasize the importance to expand community-based services and implement preventive measures—challenges encountered by many health care systems worldwide. The SMARC e-referral system’s ability to facilitate large-scale epidemiologic analysis, identify care patterns, and inform policy demonstrates the value of digital health platforms in improving coordination and transforming mental health care delivery. This study provides an exemplar that could serve as a model for other nations seeking to address mental health system challenges. This research not only informs national mental health policies but also contributes to the global understanding of mental health service delivery during extraordinary circumstances, paving the way for more resilient and adaptable health care systems.

## Supplementary material

10.2196/64257 Multimedia Appendix 1 Analysis of psychiatric e-referral data from the Saudi Medical Appointments and Referrals Centre (SMARC) system, January 2020 to December 2021. Table S1 presents the proportion of 10,033 psychiatric e-referral requests by administrative area and business units in Saudi Arabia. It shows the number and rate of requests per 10,000 people across business units and regions, allowing comparison of e-referral utilization geographically. Table S2 displays the distribution of 10,033 psychiatric e-referral reasons categorized by sex. It enables comparison of referral causes between men and women. This cross-sectional study analyzed e-referral data routinely collected in the SMARC system. Data are presented as frequency (n [%]).

## Abbreviations

BU: business unit
e-referral: electronic referral
KSA: Kingdom of Saudi Arabia
MOC: model of care
MOH: Ministry of Health
OPD: outpatient department
SMARC: Saudi Medical Appointments and Referrals Centre
