# Supplementary material for: Digital Surveillance of Mental Health Care Services in Saudi Arabia: Cross-Sectional Study of National e-Referral System Data
Source: JMIR Public Health Surveill. 2025 Jan 24;11:e64257. doi: 10.2196/64257 (PMC11785370; doi:10.2196/64257)
Supplement: Multimedia Appendix 1 — Analysis of psychiatric e-referral data from the Saudi Medical Appointments and Referrals Centre (SMARC) system, January 2020 to December 2021. Table S1 presents the proportion of 10,033 psychiatric e-referral requests by administrative area and business units in Saudi Arabia. It shows the number and rate of requests per 10,000 people across business units and regions, allowing comparison of e-referral utilization geographically. Table S2 displays the distribution of 10,033 psychiatric e-referral reasons categorized by sex. It enables comparison of referral causes between men and women. This cross-sectional study analyzed e-referral data routinely collected in the SMARC system. Data are presented as frequency (n [%]). [file publichealth-v11-e64257-s001.docx]

**Table S1:** Proportion of psychiatric e-referral requests by administrative area and business units

| **Business Units (BU)** | | **Number of requests (%)** | **Total population (%)**  **32,175,224 (100)** | **Rate per 10,000** |
| --- | --- | --- | --- | --- |
| Central BU | | 1,128 (11.24) | 9,927,927 (30.85) | 1.14 |
| Western BU | | 4,532(45.17) | 10,498,620 (32.62) | 1.16 |
| Eastern BU | | 1,220(12.16) | 5,125,254 (15.93) | 8.84 |
| Southern BU | | 1,603(15.98) | 4,021,582 (12.50) | 3.99 |
| Northern BU | | 1,550(15.45) | 2,601,841 (8.01) | 5.96 |
| **Business Units regions** | **Administrative area** | **Number of requests** | **Total population (%)**  **32,175,224 (100)** | **Rate per 10,000** |
| Central | Riyadh | 671(6.69) | 8,591,748 (26.70) | 0.78 |
|  | AL Qassim | 457(4.55) | 1,336,179 (4.15) | 3.42 |
| Western | Makkah | 2,525(25.17) | 8,021,463 (24.93) | 3.14 |
|  | Madinah | 1,714(17.08) | 2,137,983 (6.64) | 8.01 |
|  | Albaha | 293(2.92) | 339,174(1.54) | 8.63 |
| Eastern | Eastern region | 1,220(12.16) | 5,125,254 (15.92) | 2.38 |
| Southern | Aseer | 645(6.43) | 2,024,285 (6.29) | 3.19 |
|  | Jazan | 509(5.07) | 1,404,997 (4.360) | 3.18 |
|  | Najran | 396(3.95) | 592,300 (1.84) | 6.68 |
| Northern | Aljouf | 152(1.52) | 595,822 (1.85) | 2.55 |
|  | Northern Border | 753(7.51) | 373,577 (1.16) | 20.15 |
|  | Tabuk | 225(2.24) | 886,036 (2.75) | 2.53 |
|  | Hail | 473(4.71) | 746,406 (2.31) | 6.33 |

This cross-sectional study analyzed data on 10,033 psychiatric e-referrals processed through the Saudi Medical Appointments and Referrals Centre (SMARC) system. The table presents the number and rate of e-referral requests for mental health care per 10,000 population across the 5 business units (Central, Eastern, Western, Northern, and Southern) and their constituent administrative areas. This allows comparison of e-referral utilization rates across geographic regions. Data are presented as frequency (N (%)).

Abbreviation: BU=Business Unit, SMARC= Saudi Medical Appointments and Referrals Centre.

**Table S2:** Reasons of psychiatric e-referrals by sex.

| Referral Types | Sex | |
| --- | --- | --- |
|  | Male  N (%) | Female  N (%) |
| OPD | 1782 (28.87) | 966 (26.68) |
| Routine Admission | 2781 (27.78) | 1307 (36.12) |
| ER | 2780 (43.35) | 1346 (37.20) |
| Total | 6,414 (63.93) | 3,619 (36.07) |

This cross-sectional study analyzed data on 10,033 psychiatric e-referrals processed through the Saudi Medical Appointments and Referrals Centre (SMARC) system. The table presents the number and percentage of e-referrals for outpatient department (OPD), routine admission, and emergency room (ER) care, categorized by sex. This enables comparison of referral reasons between males and females in the study sample.

Abbreviations: OPD = Outpatient Department, ER = Emergency Room.
